# Versatile generation and manipulation of phase-structured light beams using on-chip subwavelength holographic surface gratings

**DOI:** 10.1515/nanoph-2022-0513

**Published:** 2023-01-03

**Authors:** Shuang Zheng, Zhenyu Zhao, Weifeng Zhang

**Affiliations:** Radar Research Lab, School of Information and Electronics, Beijing Institute of Technology, Beijing 100081, China; Key Laboratory of Electronic and Information Technology in Satellite Navigation (Beijing Institute of Technology), Ministry of Education, Beijing 100081, China; Beijing Institute of Technology Chongqing Innovation Center, Chongqing 401120, China; Chongqing Key Laboratory of Novel Civilian Radar, Chongqing 401120, China

**Keywords:** orbital angular momentum, phase-structured light beams, subwavelength grating

## Abstract

Phase-structured light beams carrying orbital angular momentum (OAM) have a wide range of applications ranging from particle trapping to optical communication. Many techniques exist to generate and manipulate such beams but most suffer from bulky configurations. In contrast, silicon photonics enables the integration of various functional components on a monolithic platform, providing a way to miniaturize optical systems to chip level. Here, we propose a series of on-chip subwavelength holographic waveguide structures that can convert the in-plane guided modes into desired wavefronts and realize complex free-space functions, including the generation of complex phase-structured light beams, arbitrarily directed vortex beam emission and vortex beam focusing. We use a holographic approach to design subwavelength holographic surface gratings, and demonstrate broadband generation of Laguerre–Gaussian (LG) and linearly polarized (LP) modes. Moreover, by assigning appropriate geometric phase profiles to the spiral phase distribution, the off-chip vortex beam manipulation including arbitrarily directed emission and beam focusing scenarios can be realized. In the experiment, directed vortex beam emission is realized by using a fabricated tilt subwavelength holographic fork grating. The proposed waveguide structures enrich the functionalities of dielectric meta-waveguide structures, which can find potential applications in optical communication, optical trapping, nonlinear interaction and imaging.

## 1 Introduction

Structured light beams, concerning the tailoring of light in amplitude, phase, and polarization, have been attracting considerable attention owing to their great potential in various applications [1, 2]. Particularly, phase-structured light beams have distinct phase distributions and resultant intensity profiles. The vortex beams carrying orbital angular momentum (OAM) are a kind of typical phase-structured light beams, which have spiral phase distributions and doughnut intensity profiles, such as Laguerre–Gaussian (LG) and Bessel–Gaussian (BG) beams [3, 4]. In the past decade, OAM light beams have seen their potential applications in high-capacity optical communications, imaging, nonlinear interaction and optical trapping [5], [6], [7], [8], [9], [10], [11], [12], [13], [14], [15]. In addition, as another mode basis, linearly polarized (LP) fiber modes are also phase-structured and widely investigated in mode-division-multiplexing (MDM) communications [16, 17], optical tweezers [18], and quantum information technology [19, 20]. Undoubtedly, both the generation and manipulation of phase-structured light beams are of vital importance for their applications.

Driven by their distinctive properties and miscellaneous applications, there have been many attempts to generate and manipulate phase-structured light beams, such as the generation, (de)multiplexing, detection, focusing and steering [21, 22]. These approaches include cylindrical lens mode converters, spiral phase plates, q-plates, spatial light modulators (SLMs), and metasurfaces [23], [24], [25], [26], [27], [28]. Especially in the past decade, due to the unprecedented flexibility in controlling electromagnetic waves, numerous metasurface structures have been demonstrated for the versatile generation and manipulation of phase-structured light beams. However, most reported approaches are based on free-space illumination, which make the optical system bulky and also require precise assembly. On the other hand, due to the increasing demands for the miniaturization of free-space optical systems down to the chip scale, free-space applications of silicon photonics and other integrated platforms have attracted much attention. Recently, a variety of photonic integrated devices based on silicon and other platforms have been demonstrated to generate and manipulate structured light beams [29], [30], [31], [32], [33], [34], [35], [36], [37], [38], [39], [40], [41], [42], [43], [44], [45], [46], [47], [48], [49], [50], [51], [52], [53], [54], [55], [56], [57], [58], [59], [60], [61]. Particularly, subwavelength waveguide structures have been considered as an attractive approach to exploit guided modes due to their unprecedented flexibility in controlling guided electromagnetic waves. For example, ultra-compact and broadband vortex beam emitters have been demonstrated by employing subwavelength surface structures [36], [37], [38]. Compared with on-chip metasurfaces [54], [55], [56], [57], [58], [59], subwavelength grating structures have smaller footprint and higher conversion efficiency. Nonetheless, until now, most demonstrated on-chip subwavelength gratings are employed for the generation of structured light beams, and few waveguide structures are reported for complicated manipulation such as directed beam emission and focusing. In practical applications, directed emission and focusing for vortex beam are indispensable. For example, in the nonlinear interaction of vortex beam, the phase matching is generally realized by adjusting the angle of the interacting beams in nonlinear materials, thus chip-scale directed vortex beam emission would be useful for system miniaturization [12, 13]. In addition, focused vortex beams could be also used for optical trapping of particles, free-space optical communication, and chip-fiber coupling applications [8, 14, 15]. In these cases, lenses are often required to focus the vortex beams, resulting in complicated optical systems, so chip-scale focused vortex beam generation is also extremely meaningful. In this scenario, compact photonic integrated waveguide structures used for versatile manipulation of phase-structured light beams deserves attention.

In this article, based on our previously reported work [37, 38], we further present a series of on-chip integrated subwavelength holographic structures for the generation and manipulation of phase-structured light beams. Based on the holographic design method, customized subwavelength holographic surface gratings are designed to realize versatile functions, including the generation of LG and LP modes, the directed emission and focusing of vortex beams. The simulation results show that subwavelength holographic gratings on strip waveguides can produce high-quality LG and LP modes over a wide wavelength range. In addition, by introducing an additional linear phase gradient into the spiral phase distribution, arbitrarily directed beam emission of vortex beam is realized. When the additional linear phase gradient along the *x*- or *y*-axis is changed from −2 × 10^6^ to 2 × 10^6^ rad/m, the offset emission angle of the generated OAM beams is tuned from −21 to 19° along the *x*-axis and from 18.8 to −20.8° along the *y*-axis but stays in the same in the other direction at 1550 nm, respectively. Furthermore, vortex beam focusing is realized by combining a parabola phase and a spiral phase, and the focal length could change from 1.3 to 4.0 μm. In the experiment, the measured results indicate that directed OAM beam emission can be realized by using a tilt subwavelength holographic surface grating. Compared with previous reports, the proposed structures enrich and extend the functionalities of meta-waveguides, which show great potential in diverse applications.

## 2 Concept and principle

**Figure 1:**
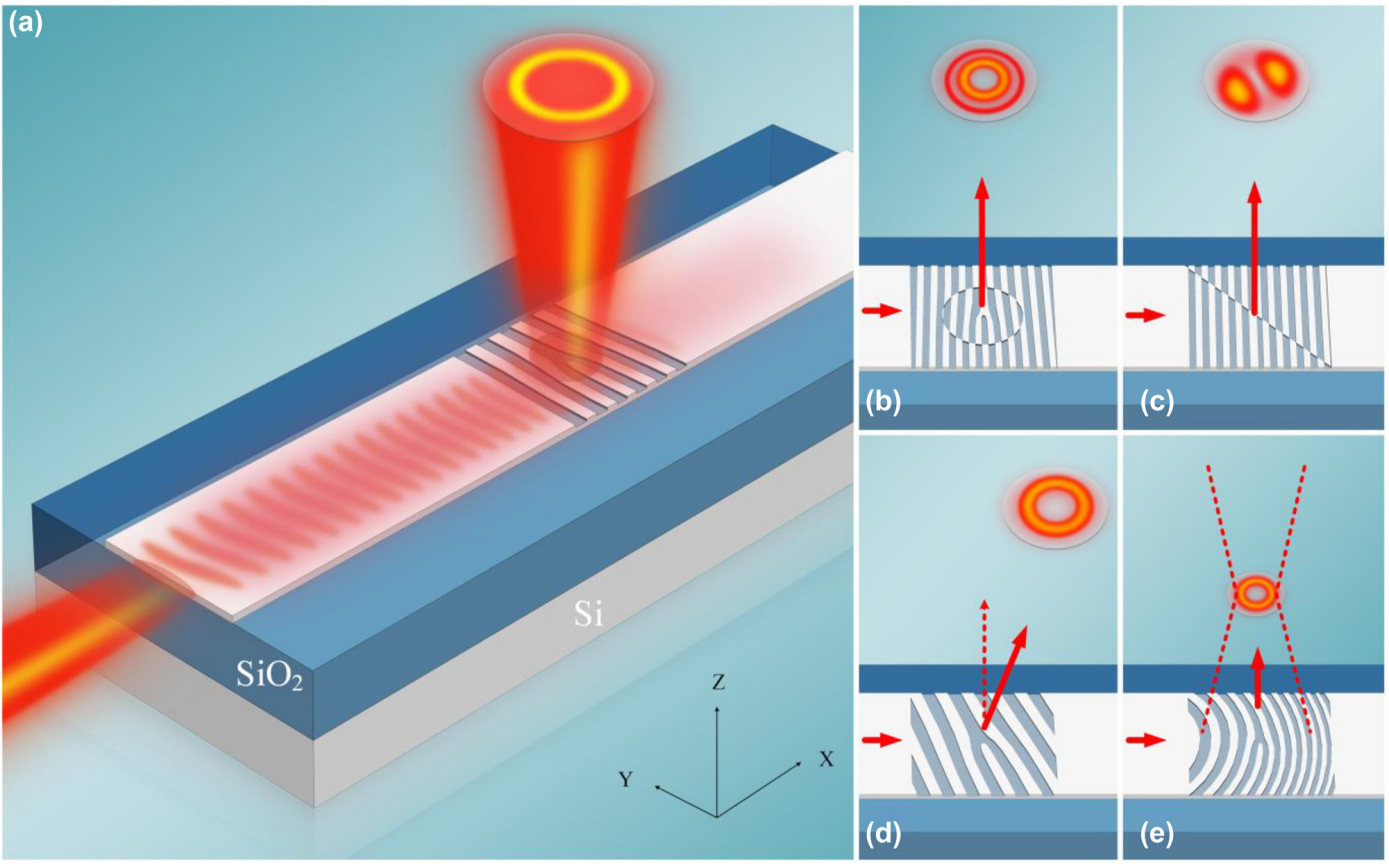
Subwavelength holographic surface gratings. (a) Schematic of the photonic integrated vortex beam generator based on holographic subwavelength fork grating. (b–e) Different holographic subwavelength fork grating structures for the phase-structured beam generation of LG and LP modes, and vortex beams manipulation of angled emission and focusing.

Figure 1(a) shows the perspective view of the phase-structured light beam generation based on subwavelength holographic surface grating structure. As can be seen, the incident fundamental mode (TE_0_) (in-plane guided mode) is coupled into free-space OAM mode (out-plane vertically emitted mode) by the subwavelength surface structure. In Figure 1(b) and (c), by tailoring the holograms of the subwavelength holographic surface grating structures, different phase-structured beams including LG and LP modes can be generated. Furthermore, by incorporating additional geometric phase profiles, the manipulation of the generated vortex beams to have different emission angles or realize the beam focusing can be realized in Figure 1(d) and (e).

To design the grating structures, the holographic patterns can be retrieved by interfering the target phase-structured light *E*_target_ = *A*exp(i*φ*) and an in-plane guided wave *E*_waveguide_ = *B*exp(i*kx*), where *A* and *B* are amplitudes of the phase-structured mode and guided wave, respectively. *φ* represents the phase distribution of the target phase-structured light. *k* is the propagation constant of the in-plane guided mode. The interference of the two waves leads to a holographic pattern, which could be written by

(1) $$Hologram={\left|E_{\text{target}}+E_{\text{waveguide}}\right|}^{2}$$

To transfer the holographic pattern onto a silicon waveguide, the hologram in Eq. (1) will be converted to a binarized pattern. Thus, when the holographic grating is illuminated with the same in-plane guided mode, the grating will modulate it and the generated out-plane field contains a term with the same phase distribution as the target phase-structured light, which is denoted as

(2) $$E_{\text{out}}=C\exp\left(i\varphi \right)$$

where *C* is the amplitude of the generated phase-structured light. As can be seen, *E*_out_ is independent of *k*_*x*_, and represents a phase-structured beam.

To realize the manipulation of the generated vortex beams, additional geometric phase profiles could be introduced to the spiral phase distribution *φ*, such as linear phase gradients and parabola phase*.* Thus, a guided mode propagating along the waveguide will be converted to the target vortex beam with directed emission or focusing property. As shown in Figure 1(b) and (e), versatile functions including the generation of LG and LP modes, the directed emission and beam focusing for vortex beams are realized by using different structures.

## 3 Simulation results

In this section, we present a series of on-chip integrated subwavelength holographic surface gratings for the free-space beam generation and manipulation of phase-structured modes. By adjusting the target phase distribution through the design strategy, the generation, directed emission and focusing of phase-structured beams can be implemented.

### 3.1 LG beams generation

LG beams form a complete basis set of orthogonal modes [62], which can be given by

(3) $$\begin{array}{ll} {LG}_{ml} & =\sqrt{\frac{2m!}{\pi \left(m+\left|l\right|\right)!}}\frac{1}{w\left(z\right)}{\left[\frac{r\sqrt{2}}{w\left(z\right)}\right]}^{\left|l\right|}\exp\left[\frac{-r^{2}}{w^{2}\left(z\right)}\right]\exp\\ & \;\times \left[il\varphi \right]\exp\left[\frac{ik_{0}r^{2}z}{2\left(z^{2}+z_{R}^{2}\right)}\right]\exp\left[-i\left(2m+\left|l\right|+1\right)\right.\\ & \;\times \left.\tan^{-1}\left(\frac{z}{z_{R}}\right)\right] \end{array}$$

where *w*(*z*) = *w*_0_[(*z* ^2^ + *z*_R_^2^)/*z*_R_^2^]^1/2^ represents the size of the light spot at the propagation distance *z*, *w*_0_ is the waist radius, *z*_R_ is the Riley distance. The light field distribution of LG_*ml*_ mode is determined by the topological charge *l* in their angular orientation and the radial nodes *m* in their amplitude profile. The value of *l* determines the spiral phase wavefront exp(i*lφ*), while *m* represents the number of nodes of the light intensity in the radial direction, which is distributed in *m* + 1 concentric circles for an LG beam with |*l*| > 0.

**Figure 2:**
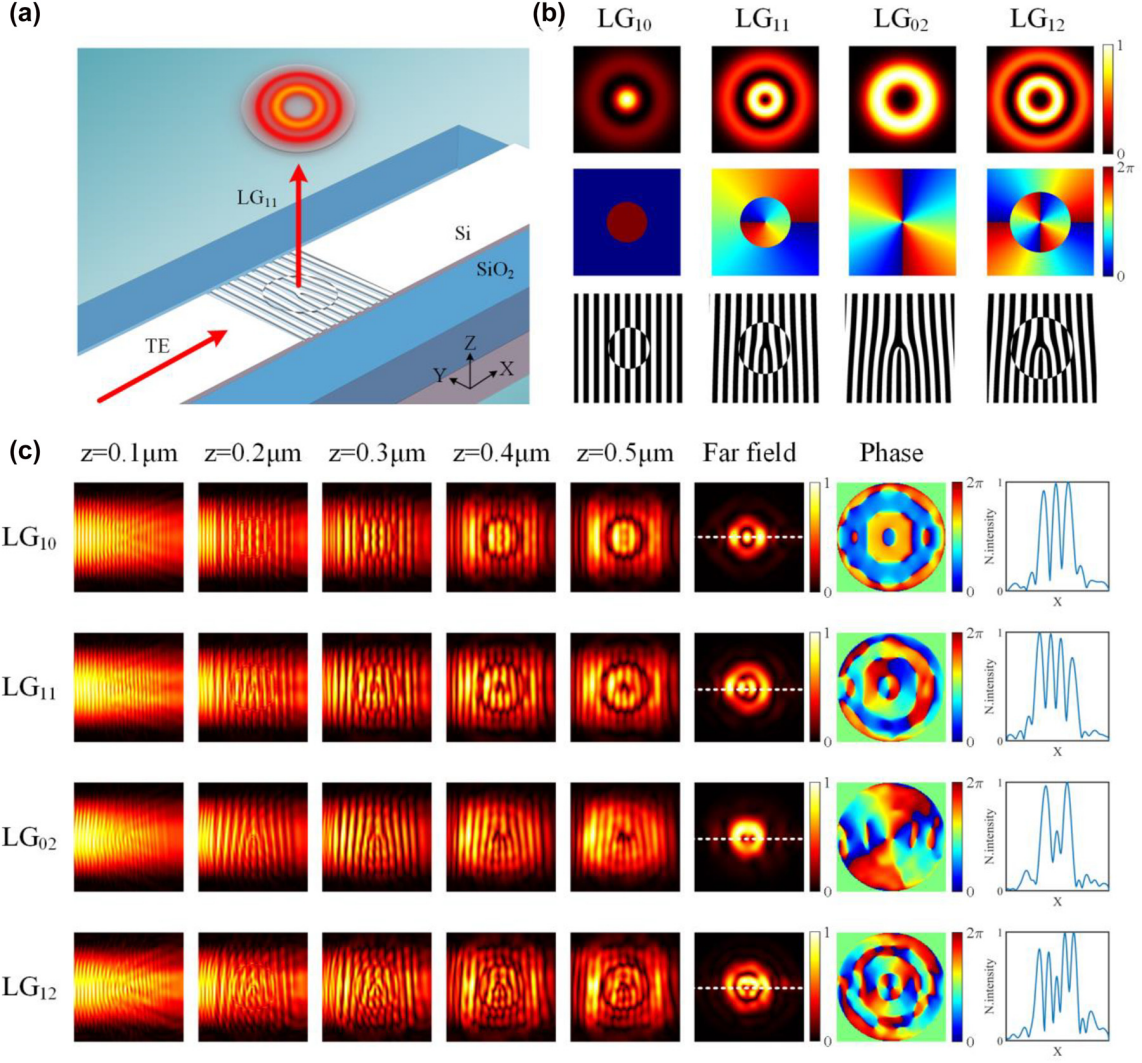
Simulation results for LG beam generation. (a) Schematic of the chip-scale broadband LG beams generator based on the holographic subwavelength grating. (b) The intensity and phase distributions of LG beams with different orders as well as the corresponding holograms. (c) Simulated electric field distributions along the waveguide at different height of the grating region, including the amplitude profiles, phase profiles, and normalized longitudinal intensity profiles.

Based on the design principle of subwavelength holographic surface gratings, we use the phase distribution of LG beams as the target phase *φ*. The schematic of the chip-scale broadband LG beam generator based on the holographic subwavelength grating is shown in Figure 2(a). As can be seen, the in-plane fundamental mode (TE_0_) in the waveguide is coupled to the out-plane LG mode by the subwavelength holographic surface grating. Figure 2(b) shows the intensity and phase distributions of four LG beams with different orders as well as the corresponding holograms. Different from the phase distribution of LG_02_ mode, LG_12_ mode has two helical phase distributions of the inner and outer rings, which have a phase difference of π. Thence, there is a dislocated grating structure distribution in the holographic image of LG_12_ mode. To provide a theoretical analysis, we simulate the proposed LG beams generator by using 3D finite-difference time-domain (3D-FDTD) method. The geometry of the subwavelength holographic surface grating region is a square with a length of 6 μm (same as the waveguide width). The propagation constant of the guided mode is determined by the effective index (∼2.7) and the input wavelength. The silicon in the grating region is etched by a depth of 70 nm and covered by the upper SiO_2_ cladding. As shown in Figure 2(c), a simulation is performed when the incident light wavelength is 1550 nm. As can be seen, the in-plane guided mode gradually evolves when propagating from the input waveguide region to the subwavelength holographic surface grating region. The light fields are diffracted into free space by the gratings, and then form stable target LG modes in the far field. The far-field intensity distributions are calculated from the near-to-far field projection in the 3D-FDTD simulation. As we can see, the generated LG beams have different angular helical phase and radial amplitude distributions in the far field. The last column in Figure 2(c) shows the normalized longitudinal intensity profiles of the generated LG modes in the far field. Since the etched gratings do not affect the transmitted waveguide mode, vortex array (LG_11_) generation could be also realized (see Supplementary Material).

**Figure 3:**
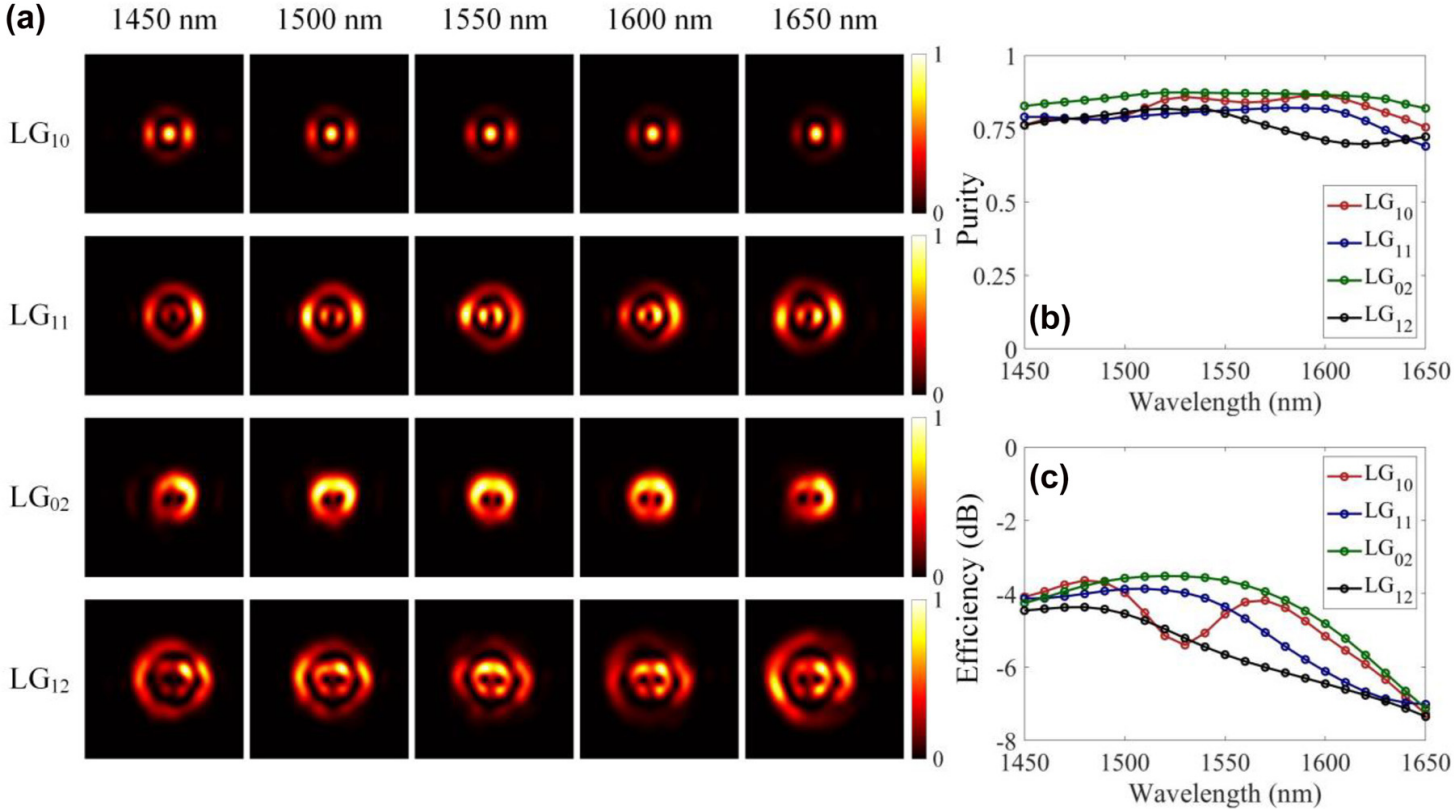
Broadband operation of the proposed LG beam generator. (a) The simulated normalized electric field intensity distribution of the generated LG beams for different topological charge l and radial node m at different wavelengths. (b, c) The simulated mode purity and emitting efficiency of the generated LG modes versus the wavelength.

We then investigate the broadband characteristics of the designed subwavelength holographic surface grating. In the simulation, the monitor for light field detection is located at a distance of 1 μm from the grating surface. Figure 3(a) shows the simulated normalized electric field intensity distributions of the generated LG beams with different topological charge *l* and radial node *m* at the wavelength of 1450 nm, 1500 nm, 1550 nm, 1600 nm, and 1650 nm. By comparing the light fields at different wavelengths, it can be found that the light fields do not degrade significantly over a wavelength range of 200 nm. Notably, the emission direction is perpendicular to the plane of grating at the designed wavelength. For an offset wavelength, there is an offset emission angle, which is determined by the period and effective index of the grating and the input light wavelength.

We use the overlap integral method to calculate the mode crosstalk of the beams by evaluating the simulated far-field intensity profiles (see Supplementary Material). As shown in Figure 3(b), for all LG mode generators, the average purities of the generated LG modes are above 0.75 over the wavelength range of 1450–1650 nm, which further indicates the broadband operating characteristic of these subwavelength devices. We also study the emitting efficiency defined as the power ratio of the emitted LG beam to the incident in-plane TE_0_ mode. As shown in Figure 3(c), the average emission efficiencies are more than −6 dB from 1450 to 1650 nm for all LG modes. Since a portion of the light is emitted downward, which enters into the substrate and induces loss, one potential way to improve the scattering efficiency is to add a gold film reflector under the SiO_2_ substrate to increase the scattering efficiency [63]. In addition, higher-order OAM modes could be also generated by designing the holographic grating (see Supplementary Material).

### 3.2 Linearly polarized (LP) modes generation

In addition to the optical vortices, LP modes also have a wide range of applications in many fields such as optical communication, rotational Doppler detection, and microscopic manipulation. The superposition of two helically phased beams with opposite values of topological charge l creates a beam cross-section with a modulated intensity of 2*l* radial petals, and the orientation of the synthesized intensity profile can be flexibly adjusted by changing the relative phase shift between the superposition modes. When introducing a phase shift to one of the spiral phases, the resulting modulated intensity will rotate around the center. The LP beams generation can be performed by employing two input ports of a vortex beam generator. However, this solution is limited by operation bandwidth and the accurate phase tuning between two light paths [37].

**Figure 4:**
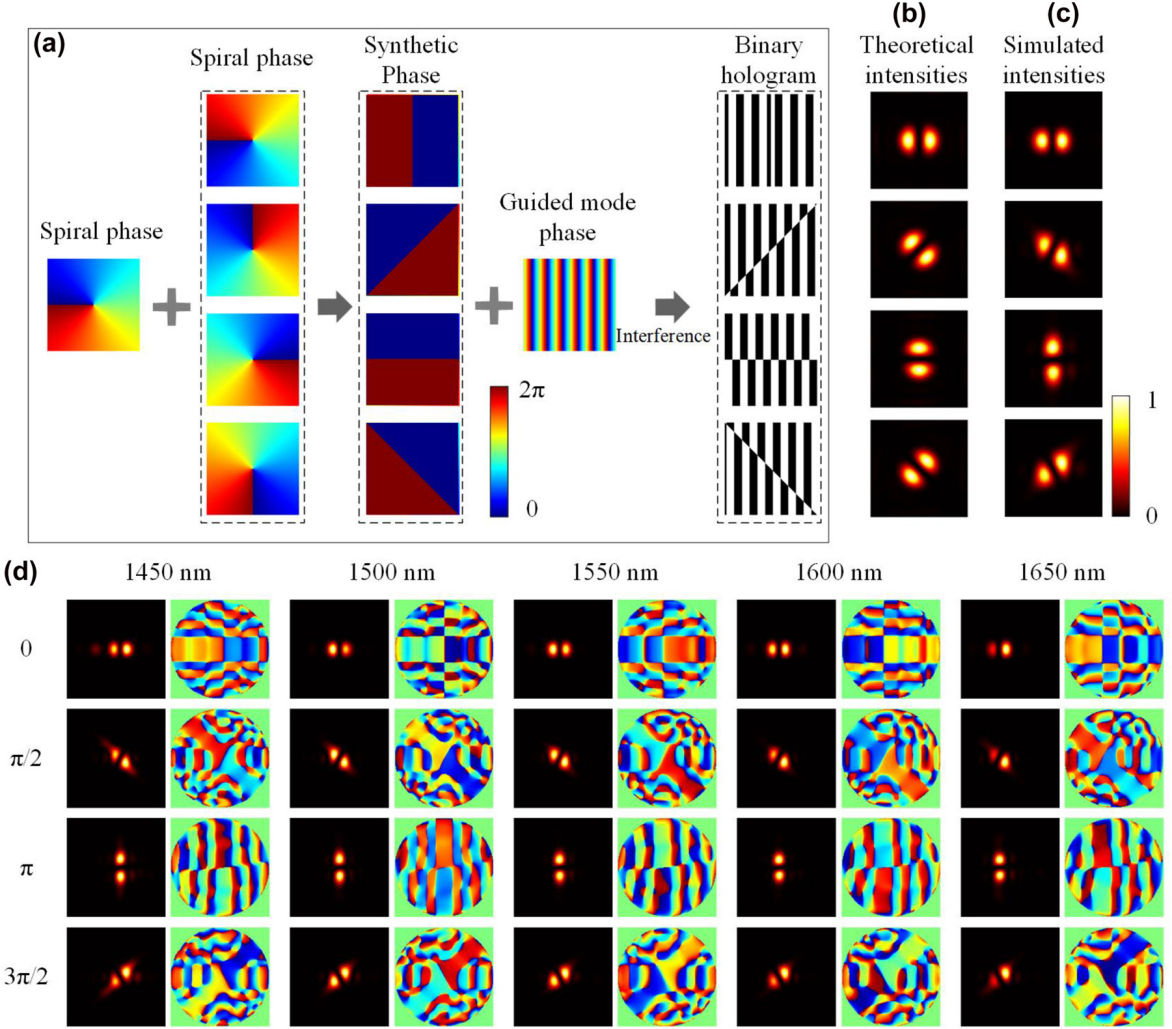
LP beam generation. (a) The design principle of the holographic subwavelength surface grating. (b, c) The theoretical and simulated intensity profiles of rotated LP beams at the wavelength of 1550 nm. (d) The simulated electric field intensity and phase profiles of the generated LP beams at 1450 nm, 1500 nm, 1550 nm, 1600 nm, and 1650 nm.

Here, we proposed a compact mode emitter for directly generating LP modes by using two superposed vortex phase distributions as the target phase *φ* according to the design strategy, and the synthesized phase profiles can be given by:

(4) $$\exp\left(i\varphi \right)=\exp\left(il\theta \right)+\exp\left(-il\theta +i\varphi_{r}\right)$$

where *φ*_*r*_ represents the relative phase shift between the vortex modes with opposite topological charges. The schematic of LP beam generation is shown in Figure 4. The design principle of the subwavelength holographic surface grating is illustrated in Figure 4(a). First, a two-lobe LP phase can be synthesized by superimposing two helical phase profiles with opposite topological charges of ±1. Then, a binary hologram can be obtained by combining a two-lobe LP phase profile and a guided mode phase. We further perform numerical simulations for different LP mode generations based on 3D-FDTD method. Figure 4(c) shows the simulated electric field intensity distributions of four different LP beams at 1550 nm by designing a 6 × 6 μm^2^ subwavelength holographic surface grating on a strip waveguide, which shows two-lobe intensity profiles. The simulated results in Figure 4(c) agree well with the theoretical ones shown in Figure 4(b). Moreover, as illustrated in Figure 4(d), the simulated results indicate that high-quality LP modes can be generated over a wide wavelength range of 1450–1650 nm. The average conversion efficiency is more than −6 dB (see Supplementary Material).

### 3.3 Directed vortex beam emission

**Figure 5:**
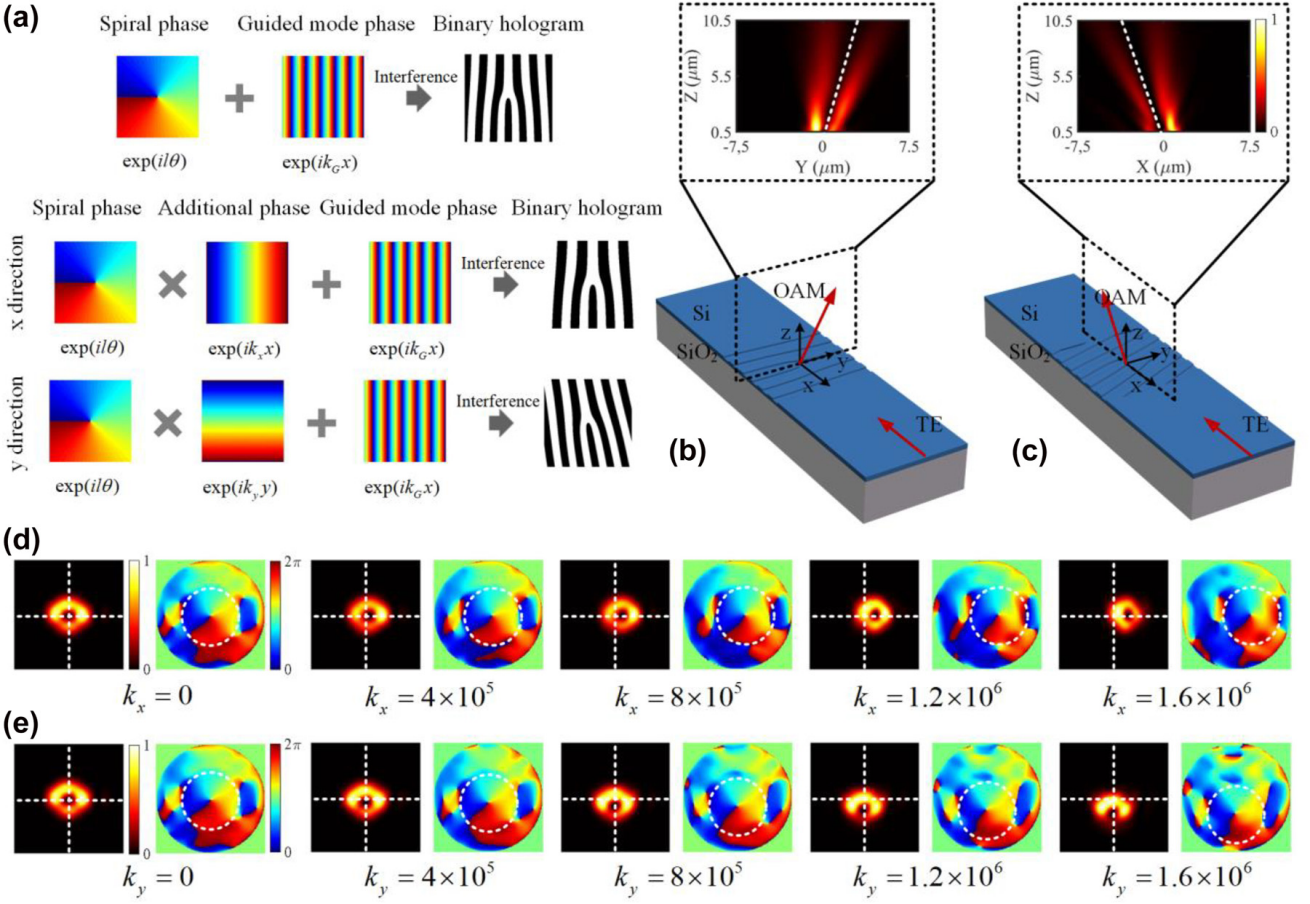
The principle and simulation results of the proposed vortex beam generator for directed emission. (a) The design principle of the subwavelength holographic fork grating. (b, c) the schematic diagram of angled emitting in the *x* and *y* directions, respectively. (d, e) The simulated normalized intensity and phase distribution of the angle emitting vortex beams with respect to the additional phase *k*_*x*_ and *k*_*y*_, respectively.

It is known from Fourier optics that a lens works as a phase-type diffractive device, and the diffracted light is determined by its phase distribution. In general, a sloped isophase surface produced by a linear phase gradient deflects the beam and the deflection angle is proportional to the phase gradient. On the basis of this, an additional linear phase gradient in the *x*- or *y*-direction is introduced to realize directed vortex beam emission. The design principle and simulation results are shown in Figure 5. As can be seen from Figure 5(a), additional linear phase gradients in the *x*- or *y*-direction are introduced to the spiral phase, and the synthesized phase profiles can be given by:

(5) $$\exp\left(i\varphi \right)=\exp\left[i\left(l\theta +k_{x}x\right)\right]$$

(6) $$\exp\left(i\varphi \right)=\exp\left[i\left(l\theta +k_{y}y\right)\right]$$

**Figure 6:**
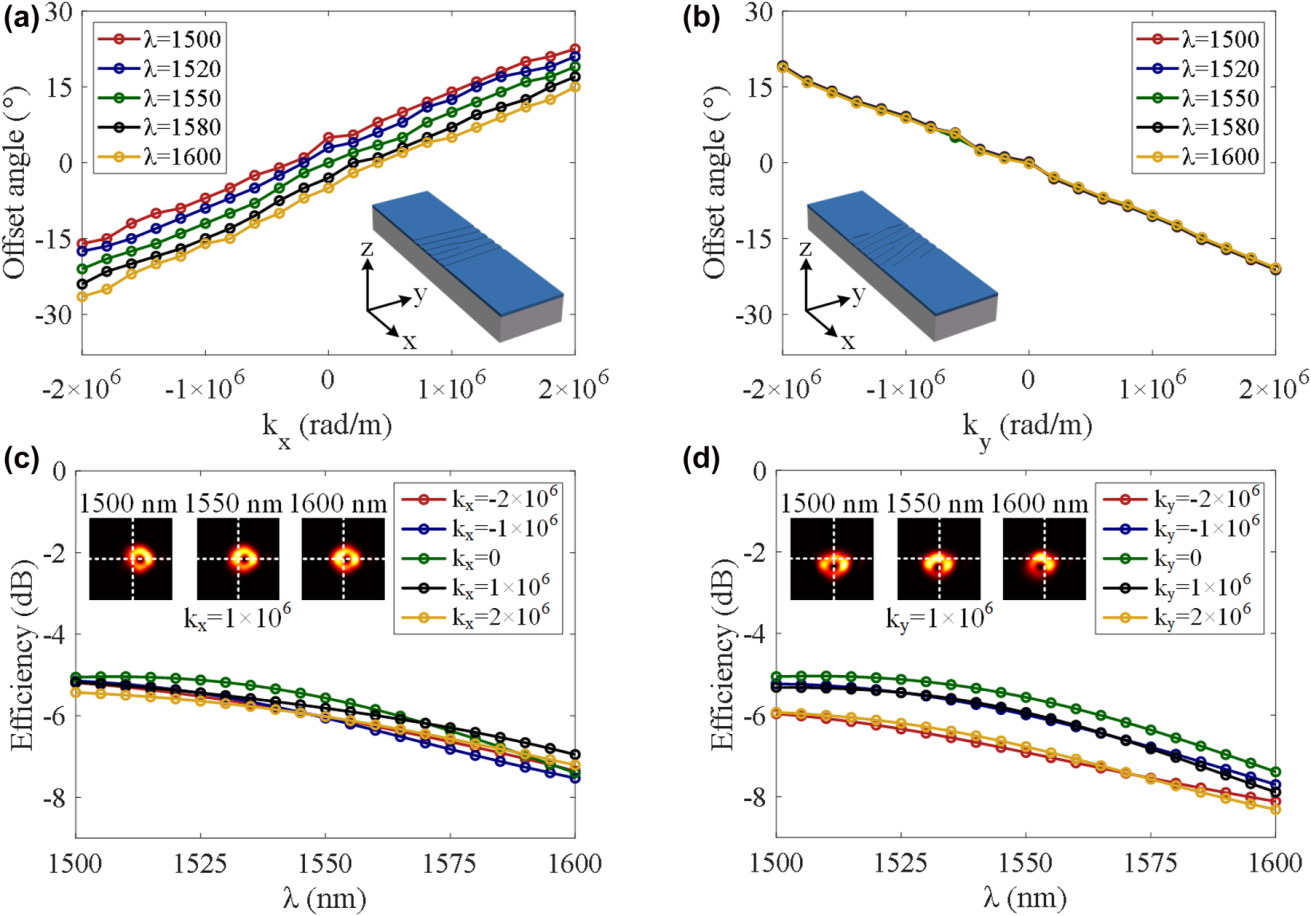
Simulated offset angle and scattering efficiency. (a, b) Offset angle versus additional linear phase gradient in the *x*-axis and *y*-axis at different wavelengths. (c, d) Simulated efficiency versus wavelength with different additional linear phase gradients in the *x*- and *y*-axis, respectively.

Based on the holographic method, the binary fork holograms can be produced by combining the target OAM mode with the in-plane guided mode. The schematic diagram is illustrated in Figure 5(b) and (c), directed OAM beam emission could be realized by engineering the grating design. As can be seen, customized gratings are implemented by shallow-etching corrugations on top of silicon waveguides. The silicon in the grating region is etched by a depth of 70 nm and covered by the upper SiO_2_ cladding. When the in-plane fundamental TE_0_ guided mode is diffracted by the grating, the out-plane vortex beam could be generated with a specified emission angle along the *x*- or *y*-axis. The insets of Figure 5(b) and (c) give the intensity profiles of the *y*–*z* and the *x*–*z* cross sections of the emitted OAM beams, and the dashed lines represent the emission direction, indicating that the generator could implement directed emission. To provide a more detailed theoretical analysis, the proposed OAM generator is simulated with 3D-FDTD method. The additional phase gradient in the *x*- or *y*-direction is dependent on the value of *k*_*x*_ or *k*_*y*_, respectively. The geometry of the holographic fork grating region is a rectangle with a length of 3.4 μm and a width of 4.2 μm (same as the waveguide width). Figure 5(d) and (e) shows the simulated far-field electric field intensity distributions. As can be seen, the offset emission angle becomes larger as *k*_*x*_ or *k*_*y*_ increases. As shown in Figure 6(a), when the additional linear phase gradient along the *x*-axis is changed from −2 × 10^6^ to 2 × 10^6^ rad/m, the offset emission angle of the generated OAM beams is tuned from −21 to 19° along the *x*-axis at the wavelength of 1550 nm. The tuning of the working wavelength would also affect the emission angle along the *x*-axis, which provide an opportunity to steer the OAM beam dynamically by adjusting light wavelength. As shown in Figure 6(a), the emission angle can be further tuned by approximately 12° along the negative *x*-axis when the wavelength is adjusted from 1500 to 1600 nm.

In Figure 6(b), when the additional linear phase gradient along the *y*-axis is changed from −2 × 10^6^ to 2 × 10^6^ rad/m, the offset emission angle of the generated OAM beams is tuned from 18.8 to −20.8° along the *y*-axis at the wavelength of 1550 nm. The insets in Figure 6(a) and (b) show the structural designs of the vortex beam emitters used for directed emission. It is worth noting that the emission angles are slightly asymmetrical when input from the opposite port, which is since the holographic surface grating is not perfectly symmetrical. Figure 6(c) and (d) show the emission efficiencies of the vortex beams with different additional linear phase gradients (−2 × 10^6^ rad/m, −1 × 10^6^ rad/m, 0 rad/m, 1 × 10^6^ rad/m, and 2 × 10^6^ rad/m). It can be clearly seen that the emission efficiency remains stable with the change of *k*_*x*_ in Figure 6(c), and the emission efficiency is reduced with the increase of *k*_*y*_ in Figure 6(d). Therefore, the emission angle of the OAM beams from the customized holographic subwavelength fork grating can be precisely controlled in two dimensions by engineering the additional linear phase gradients. In Figure 5, the emitted vortex beams become more and more asymmetric with the increased phase gradient along the *x*- or *y*-axis. One can see that the calculated mode purity decreases with the increase of emission angle (see Supplementary Material). To compensate this degeneration, the grating structure parameters could be further improved by using inverse design method and many optimization algorithms.

### 3.4 Vortex beam focusing

**Figure 7:**
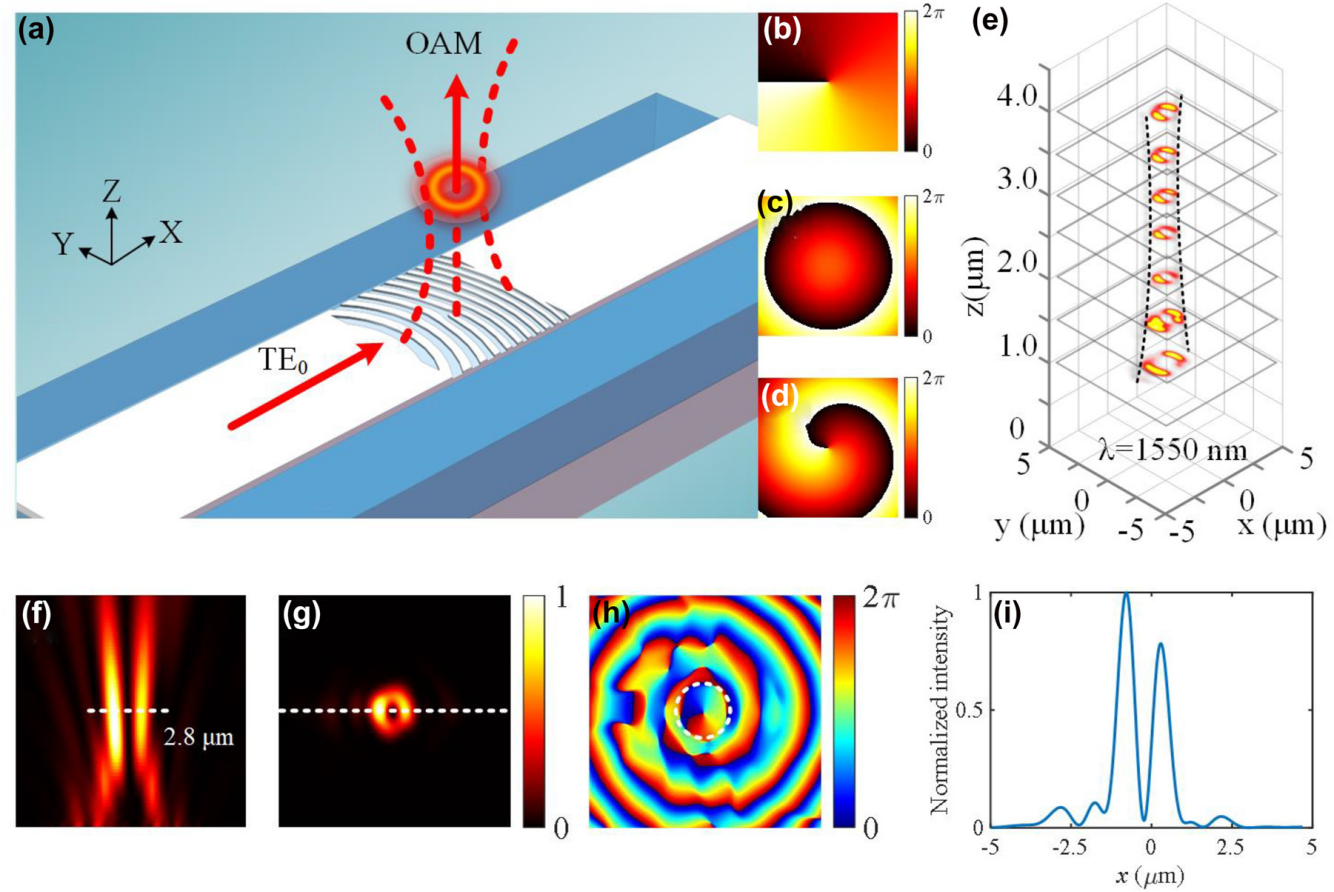
Focused vortex beam generation. (a) Schematic of the focused vortex beam generation. (b–d) The spiral phase, parabola phase, and superimposed phase distributions. (e) The simulated intensity distributions on the different focal planes at the wavelength of 1550 nm. (f) The simulated intensity profiles on the *xz* plane. (g, h) The simulated intensity and phase profiles of the generated OAM_-1_ beam on the *xy* plane on the focal plane. (i) Normalized intensity spectra of the focused OAM_−1_ mode along the *x*-axis for the figure (g).

The results above have already shown the ability of the subwavelength holographic fork grating to achieve the directed emission of the vortex beam. Similarly, focused optical vortex generation can be achieved by introducing a parabola phase distribution. Figure 7(a) shows the schematic diagram of the proposed focused vortex beam generator. Figure 7(b)–(d) show the spiral phase distribution *φ*_1_ = *lθ* along the azimuthal direction that produces the OAM beam, the parabola phase distribution $\varphi_{2}=k\sqrt{f^{2}+r^{2}}$ along the radial direction that focuses the OAM beam on the desired focal length, and the superimposition of *φ*_1_ and *φ*_2_. The synthetic phase *φ* can be expressed by [22]:

(7) $$\varphi \left(r,\theta \right)=l\theta +k\left(f-\sqrt{f^{2}+r^{2}}\right)$$

where *f* is the focal length, *l* represents topological charge, *r* is the radial distance and *θ* refers to the azimuthal angle at the transverse plane. To obtain the subwavelength holographic grating, the superimposed phase is used as the target phase *φ* to interfere with a guided wave. The geometry of the grating is a square with a length of 6 μm (same as the waveguide width). In the structure design, the propagation constant of the guided mode is determined by the effective index (∼2.7) and the input light wavelength. The silicon in the grating region is etched by a depth of 70 nm and covered by the upper SiO_2_ cladding.

**Figure 8:**
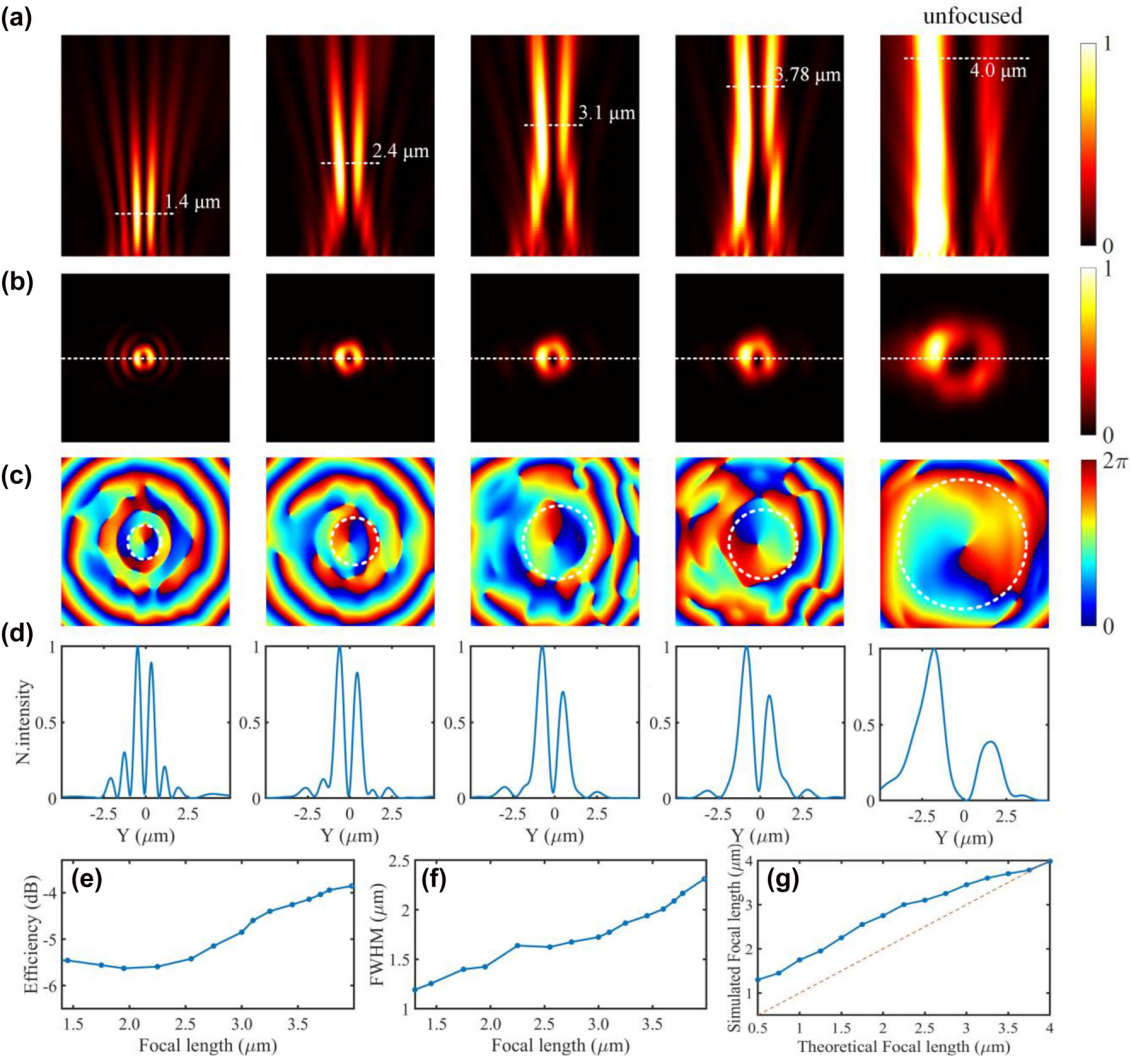
Simulated results for focused vortex beam generation. (a) The simulated intensity profiles along the *x*-axial plane. (b–d) The intensity, phase, and normalized longitudinal electrical field profiles of the OAM beam on the focal planes. (e–f) The scattering efficiency and the full width at half maximums (FWHWs) versus the focal length. (g) The theoretically designed focal length and the simulated focal length.

Figure 7(e) shows the simulated intensity distributions on different focal planes at the wavelength of 1550 nm when the holographic subwavelength grating is illuminated with an in-plane guided mode. As shown in Figure 7(e), the two-dimensional intensity slices along the *z*-direction show the evolution of the generated vortex beam, which have different spot sizes at different heights from the grating surface. More detailed simulation results are shown in Figure 7(f)–(i) with the theoretical focal length of *f* = 2 μm. As shown in Figure 7(f), the intensity profile on the *xz* plane shows that the emitted OAM beam is focused at 2.8 μm from the grating surface and the horizontal dashed line represents the focal plane. Figure 7(g) and (h) show the simulated intensity and phase profiles of the generated OAM beam on the focal plane, respectively. Figure 7(i) shows the cut along the *x*-axis of the generated focusing vortex beam in Figure 7(i). Then, the focused OAM beam generation with different focusing lengths are simulated. Columns 1–4 of Figure 8(a)–(d) show the simulated intensity profiles on the *xz* plane, the intensity and phase profiles on the *xy* plane, and normalized longitudinal profiles on the focal plane with different focal lengths, respectively. Column 5 of Figure 8(a)–(d) shows the unfocused case. As can be seen, the spot size on the focal plane increases synchronously with increasing focal length. Besides, we also characterize the quality of the focal spots. As shown in Figures 8(e) and (f), one can see that the scattering efficiency and the full width at half maximums (FWHWs) also show an increasing trend when the focal length changes from 1.3 to 4.0 μm. Figure 8(g) shows the theoretical focal length and the simulated focal length. It can be seen that as the focal length increases, the difference between them becomes smaller.

## 4 Experimental results

**Figure 9:**
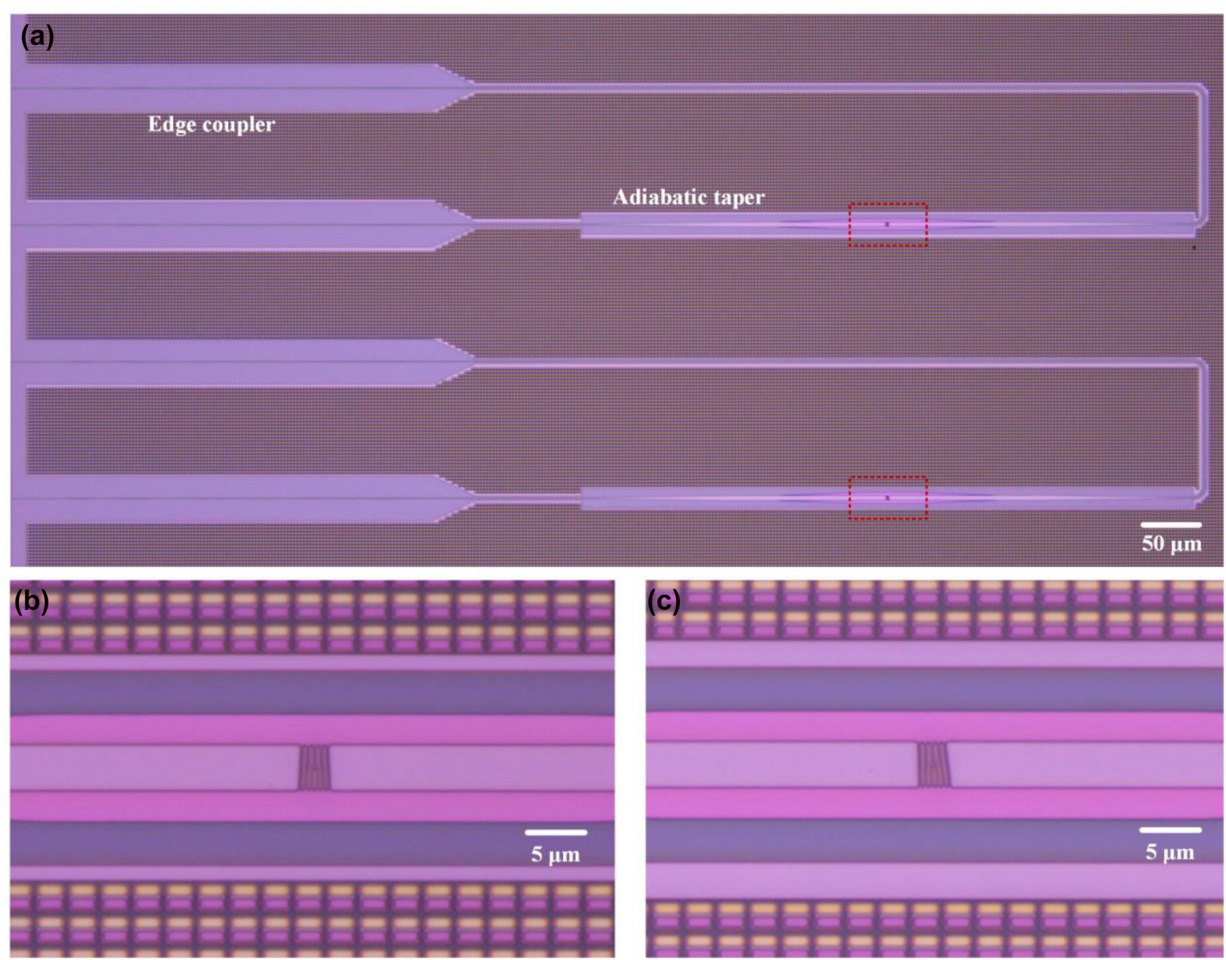
Fabricated devices. (a) Measured optical microscope images of the devices. (b, c) Measured optical microscope images of conventional and tilt subwavelength holographic surface fork gratings.

In the experiment, we fabricate a tilt subwavelength holographic surface fork grating to generate OAM_±1_ modes and manipulate the emission directions. The proposed device is fabricated on a silicon photonic platform through multi project wafer (MPW) in CUMEC (China Cor) with standard SOI processes of 248-nm deep ultraviolet (DUV) lithography and inductively-coupled plasma dry etching. The process starts with a 220-nm-thick top silicon layer and a 2-μm-thick buried oxide layer. In our design, the additional phase gradient is *k*_*y*_ = 8 × 10^5^ rad/m, which corresponds to a theoretical emission angle of 8°. The structural parameters including length, width, and depth are 3.4 μm, 4.2 μm, and 70 nm, respectively. For comparison, a conventional subwavelength holographic surface fork grating without additional linear phase gradient is also fabricated and characterized. Figure 9 shows the measured optical microscope images of the fabricated subwavelength holographic surface fork gratings etched on silicon waveguides. In Figure 9(a), the layouts of holographic fork gratings connected by adiabatic tapers are shown. To guarantee the single-mode transmission in the multimode silicon waveguide, a polarization controller is used to adjust the polarization state of light in the input fiber, and a fiber-chip edge coupler is designed for in-plane single-mode coupling. A taper is also used to change the waveguide width from 500 nm to 4.2 µm. Thus, higher-order modes are not excited. The measured optical microscope images of the fabricated conventional and tilt fork gratings are shown in Figure 9(b) and (c). One can see that the designed subwavelength surface structures are formed on top of shallow-etched silicon waveguides.

**Figure 10:**
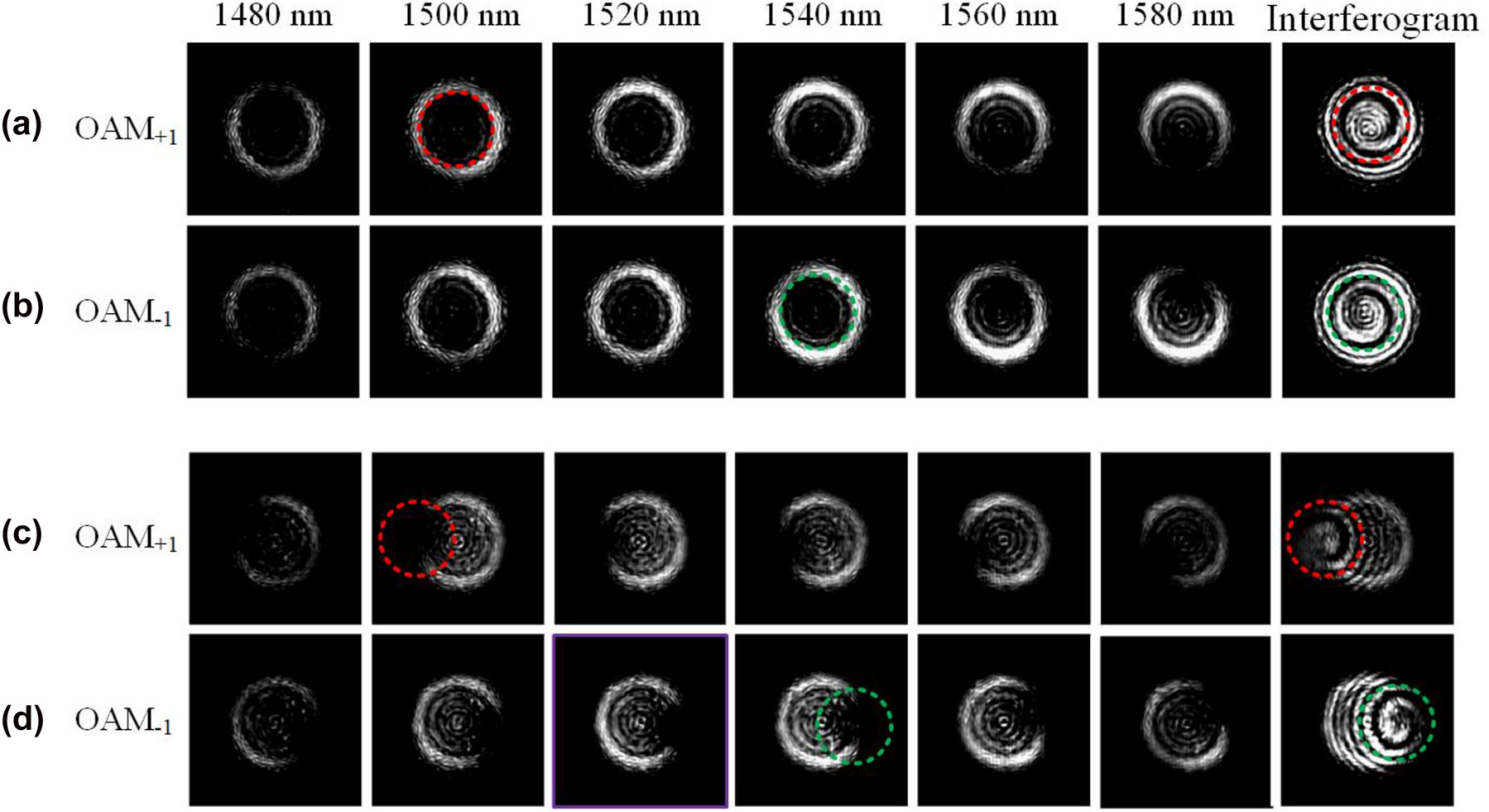
Measured far-field intensity profiles and interferograms for (a, b) conventional subwavelength holographic surface fork grating and (c, d) tilt subwavelength holographic surface fork grating.

For comparison purposes, we first demonstrate the generation of broadband OAM modes using the fabricated conventional subwavelength holographic fork grating. Figure 10 shows the measured results (intensity profiles and interferograms) for the generation of OAM_±1_ modes from 1480 to 1580 nm. One can confirm the order (magnitude and sign) of the generated OAM modes from the number of twists and twist direction of the interferograms. Figure 10(a) and (b) shows the measured results for the fabricated conventional subwavelength holographic surface fork grating. It is worth noting that the emission angle can be changed by adjusting the working wavelength. As the input light wavelength changes from 1480 to 1580 nm, the emitted OAM modes are steered on the *xz* plane. In the experiment, the offset emissions from the fork grating are collimated by a ×10 objective lens, which leads to incomplete circular light fields. As shown in Figure 10(a), one can clearly see the light field evolution due to the offset emission. The vertical emission occurs around 1530 nm, and the offset angles at 1480 and 1580 nm are just the opposite.

We then measure the fabricated tilt subwavelength holographic fork grating. The measured far-field intensity profiles and interferograms for the tilt subwavelength holographic surface fork grating are shown in Figure 10(c) and (d). As can be seen, the output OAM beams have opposite topological charge values (−1, 1) between the input optical signals from left to right and from right to left. The incomplete circular light fields are clearly observed, which indicate a tilt emission in the *y*-direction (perpendicular to the waveguide direction). In Figure 10(c), it can be seen from the interferogram that the optical singularity is significantly deviated from the center of the circular light field due to a large tilt angle. Compared with the measured light fields in Figure 10(c), the measured output OAM_–1_ beam in Figure 10(d) has an opposite emission angle due to an opposite additional linear phase gradient. The experimental results indicate that the OAM_±1_ beams are steered on the *xz* plane by tuning the working wavelength, and the directed emission on the *yz* plane can be realized by introducing an additional linear phase gradient in the design. A single lens with NA = 0.54 is further used to improve the image quality (see Supplementary Material). As shown in Figure S4, it can be seen that clear patterns are obtained. We also measure the tilt interferograms of the generated OAM beam and reconstruct the phase distributions by Fourier transform method. The calculated phase purity of the generated OAM mode is shown in Figure S5. The measured scattering efficiency of the tilt fork grating is shown in Figure S6.

Table 1 shows the comparison between the reported works and our work. As can be seen, the metasurface integrated on waveguide can provide more freedom (amplitude/phase or polarization) for the structure design, and enables versatile functionalities, including focusing, vortex beam generation, and hologram. However, the scattering efficiency is greatly limited. Also, the hybrid integration may increase the fabrication complexity. In this work, the customized gratings are implemented by shallow-etching corrugations on top of silicon waveguides, which are etched by a depth of 70 nm and covered by the upper SiO_2_ cladding. By comparison, the fabrication process is simpler and fully compatible with CMOS technology, and the holographic gratings have a bandwidth of >200 nm and higher scattering efficiency (see Supplementary Material). In addition, compared with our previous work, an additional geometric phase is introduced into the structure design, which could further enrich the functionality, such as the generation and manipulation of phase-structured light beams, which have not been demonstrated before. Some future perspectives toward a more robust optical vortex chip could be considered as follows:

(1) Devices with higher efficiency. The theoretical average scattering efficiency over 200 nm bandwidth exceeds 25%. In the experiment, due to the imperfect etching depth and taper design, the measured scattering efficiency is smaller than the simulated value. In the future, one potential way to improve the scattering efficiency is to add a gold film reflector under the SiO_2_ substrate to increase the scattering efficiency. And the waveguide structures including edge coupler and adiabatic taper can be further optimized. It could be noticed that a favorable performance could be also achieved even when generating high-order OAM modes in the presence of the trade-off relationship between the efficiency and the purity.
(2) Devices supporting high-order OAM modes and high-order waveguide modes (such as TE_1_ and TM_0_ mode). Superimposed gratings that support both polarization/space multiplexed modes might provide more multiplexed channels. Besides, multiport waveguide crossing with more than two/four input ports might offer advanced functions [63].
(3) Improvement of device performance and function. The functions demonstrated in this work includes versatile generation and manipulation of phase-structured light beams, including the generation of OAM/LP modes, arbitrarily directed vortex beam emission and vortex beam focusing. Although relatively high purity and high efficiency are achieved in the present design, one may expect higher purity and more complicated functions by future optimization. In our design, the designed structures have large index contrast between the core Si and the cladding SiO_2_, resulting in relatively high conversion efficiency and compact footprint. The presented silicon surface structure is suitable for the manipulation of phase-structured light fields with continuous uniform phase distribution, but may be difficult for implementing holographic images. For more complicated functions such as arbitrary holographic images, integrated platforms with small index contrast will be very helpful, such as SiN and polymer. And a complex phase grating (not binary phase) enabled by 3D structure design will be helpful, which can be fabricated by using gray-scale electron-beam lithography [64]. In addition, high-order OAM modes having multiple spatially separated singularities could be also optimized by using various algorithms [65].

**Table 1:** Comparison of the reported structures.

Method	Material	Size	Bandwidth	Efficiency	Functionality
Metasurfaces	Si on LNOI [56]	50 µm	>200 nm	∼10%	Focusing, vortex beam generation, hologram
	Si on Si_3_N_4_ [57]	360 µm	>100 nm	9.2%	Hologram
	Polymer on Si_3_N_4_ [58]	400 µm	>60 nm	/	Focusing, vortex beam generation, hologram
	a-Si on Si [59]	40 µm	/	17%	Focusing, hologram
Holographic gratings	ZEP on SiO_2_ [60]	300 µm	/	<1%	Hologram
	Si [38]	3.6 µm	>130 nm	∼20%	Vortex beam generation
This work	Si	3.4/6 µm	>200 nm	>25%	OAM/LP mode generation, OAM focusing, directed emission

## 5 Conclusions

In summary, we propose and demonstrate a series of on-chip integrated holographic subwavelength gratings for the generation and manipulation of phase-structured light beams. The operation principle relies on the mode coupling from the in-plane guided mode to the out-plane free-space phase-structured mode by the customized subwavelength surface structures. The simulation results show that the designed subwavelength holographic waveguide structures can generate LG and LP modes over a wide wavelength range. In addition, directed vortex beam emission is realized by introducing an additional linear phase gradient. When the additional linear phase gradient along the *x*- or *y*-axis is changed from −2 × 10^6^ to 2 × 10^6^ rad/m, the offset emission angles of the generated OAM beams are tuned from −21 to 19° along the *x*-axis and from 18.8 to −20.8° along the *y*-axis but stays in the same in the other direction at the wavelength of 1550 nm, respectively. Furthermore, vortex beam focusing is achieved by introducing an additional parabola phase profile, and the focal length can be tuned from 1.3 to 4.0 μm. In the experiment, we demonstrate directed vortex beam emission with the fabricated tilt fork grating. The proposed waveguide structures enrich the functionalities of phase-structured light beam generators, which may find potential applications in optical communication, light–matter nonlinear interactions and optical trapping.

## Supplementary Material

Supplementary Material Details
