# Supplementary material for: Versatile generation and manipulation of phase-structured light beams using on-chip subwavelength holographic surface gratings
Source: Nanophotonics. 2023 Jan 3;12(1):55–70. doi: 10.1515/nanoph-2022-0513 (PMC11501405; doi:10.1515/nanoph-2022-0513)
Supplement: Supplementary file 1 — Supplementary Material Details [file j_nanoph-2022-0513_suppl.docx]

Supplementary Material for:

Versatile generation and manipulation of phase-structured light beams using on-chip subwavelength holographic surface gratings

*Shuang Zheng, Zhenyu Zhao, and Weifeng Zhang^*^*

*^1^Radar Research Lab, School of Information and Electronics, Beijing Institute of Technology, Beijing 100081, China*

*^2^Key Laboratory of Electronic and Information Technology in Satellite Navigation (Beijing Institute of Technology), Ministry of Education, Beijing 100081, China*

*^3^Beijing Institute of Technology Chongqing Innovation Center, Chongqing, 401120, China*

*^4^Chongqing Key Laboratory of Novel Civilian Radar, Chongqing 401120, China*

[**weifeng.zhang@bit.edu.cn*](mailto:*weifeng.zhang@bit.edu.cn)

1. Simulated mode transmission process and vortex array generation


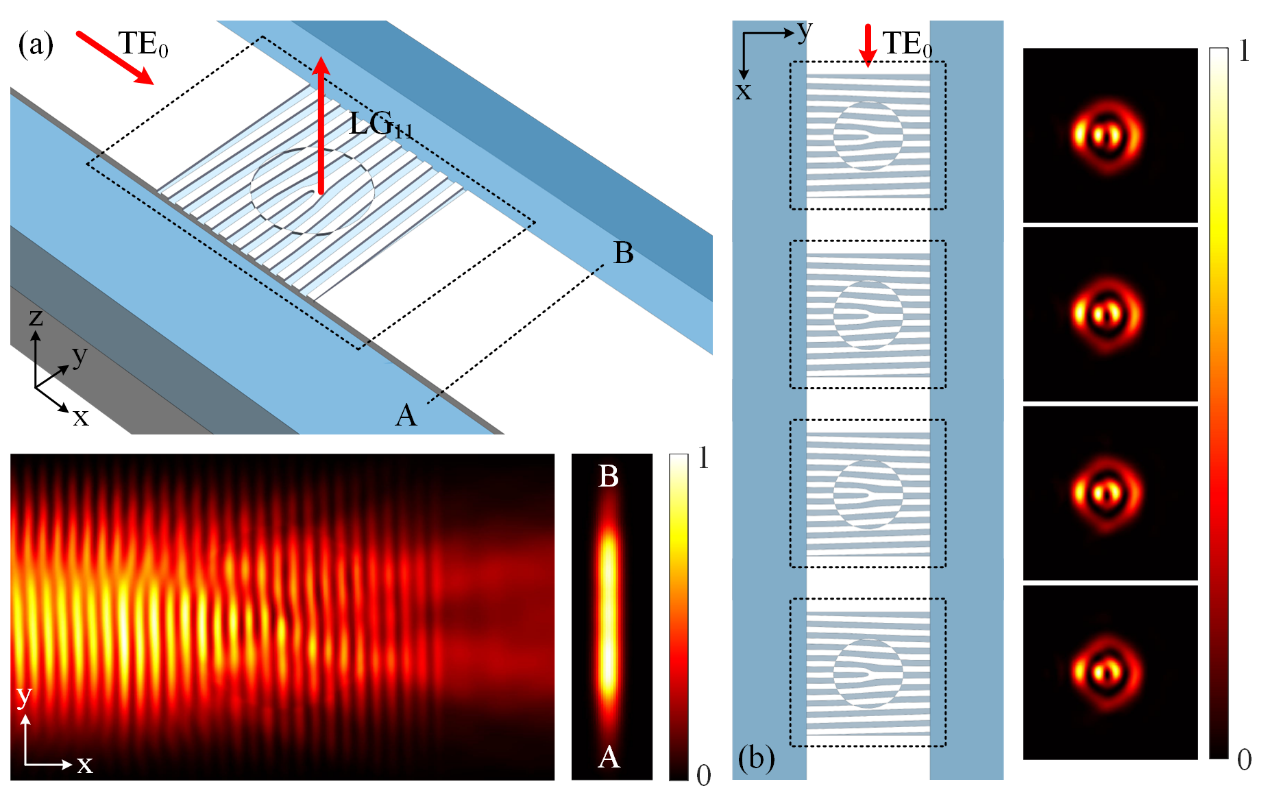


**Figure S1.** (a) The simulated mode transmission process. (b) Four fork gratings are used for vortex array generation.

The etched silicon gratings can convert the fundamental TE_0_ mode to phase-structured light beams, and won’t affect the transmitted waveguide mode. As can be seen from Fig. S1(a), the guided mode is still a TE_0_ mode. And vortex array could be also realized by integrating more subwavelength gratings, which further indicates the single-mode transmission. As shown in Fig. S1(b), four fork gratings etched on silicon waveguide are used for vortex array (LG_11_) generation. One can see that the emitted light fields are almost identical patterns and different transmission (0.38, 0.14, 0.05, 0.019).

2. Calculation of mode purity and mode crosstalk

We used the overlap integral method to calculate the mode purity of the beams by evaluating the simulated far-field intensity profiles. The overlap integral is introduced as follows:

$p=\frac{\left|\int E_{0}^{*}(x,y,z)E_{t}(x,y,z)dxdydz\right|^{2}}{\int\left|E_{0}^{*}(x,y,z)\right|^{2}dxdydz\int\left|E_{t}(x,y,z)\right|^{2}dxdydz}$ (S1)

where E_0_(x, y, z) and E_t_(x, y, z) are the complex amplitudes of the obtained mode and target mode, respectively. The mode purity is highly dependent on the size of the target beam. Thus, we calculate the mode purity with different target beam size in the simulation. As can be seen in Fig. S2, when the size of the target beam changes from small to large, a maximum purity value could be obtained.


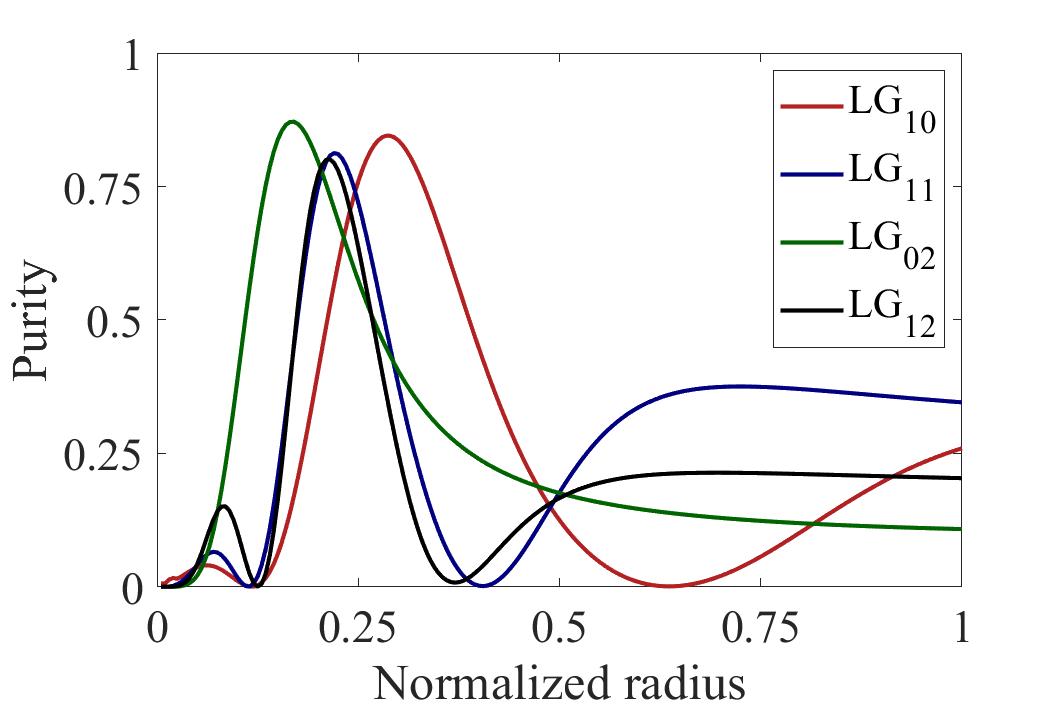


**Figure S2.** The calculated mode purity with the change of the set beam sizes.


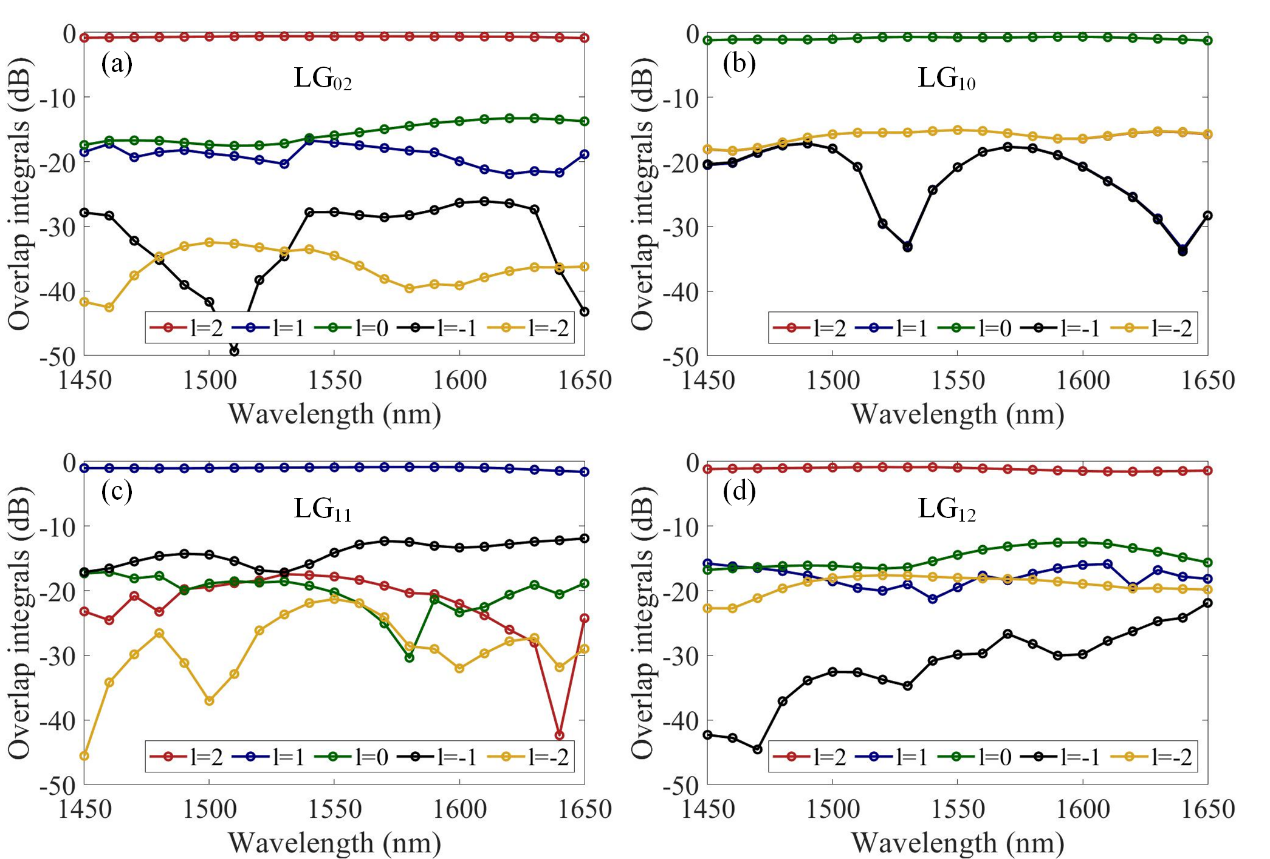


**Figure S3.** The evolution diagram from the function to the binary grating for directed OAM beam generation.

In practical applications, high-purity mode generation is highly desired. For example, in mode-division multiplexing (MDM) systems, high purity also means the low mode crosstalk. Thus, the calculation of mode crosstalk is more important. As can be seen in Fig. S3, the average extinction ratios of four different LG beams are more than 15 dB, which could support high-speed MDM data transmission.

3. Additional results for directed OAM beam emission

The interference patterns in Fig. 10 are not clear, this is because a ×10 objective lens (Mitutoyo, NIR, NA=0.26) is used in the experiment. Since the fabricated fork grating has a 4.2×3.4 µm^2^ footprint, a lens with larger NA is needed for the measurement. A single lens with NA=0.54 is used to improve the image quality. As shown in Fig. S4, it can be seen that clear patterns are obtained. We also measure the tilt interferograms of the generated OAM beam and reconstruct the phase distributions by Fourier transform method. The calculated phase purity of the generated OAM mode is shown in Fig. S5. The measured scattering efficiency of tilt fork grating is shown in Fig. S6. One can see that the measured scattering efficiency is smaller than the simulated values. On one hand, the etching depth of the fork grating is less than 70 nm, which leads to a smaller scattering efficiency. On the other hand, the adiabatic tapering (length >1 mm) loss cannot be ignored. In the future, one potential way to improve the scattering efficiency is to add a gold film reflector under the SiO_2_ substrate to increase the scattering efficiency. And the waveguide structures including edge coupler and adiabatic taper can be optimized.


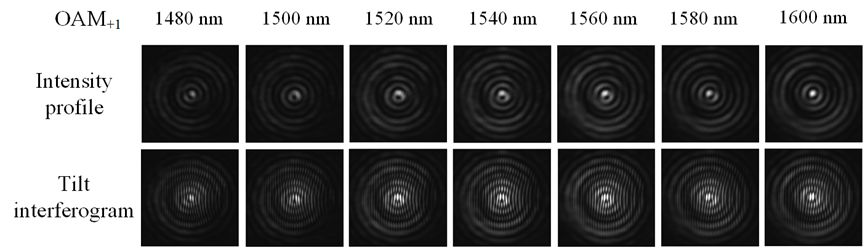


**Figure S4.** The measured far-field intensity profiles of tilt fork grating.

**Figure S5.** The calculated phase purity of the generated OAM mode.

**Figure S6.** The measured scattering efficiency of tilt fork grating.

4. Additional results for LP beam emission

As illustrated in Fig. S7, the average conversion efficiency is more than -6 dB over a wide wavelength range of 1450-1650 nm.


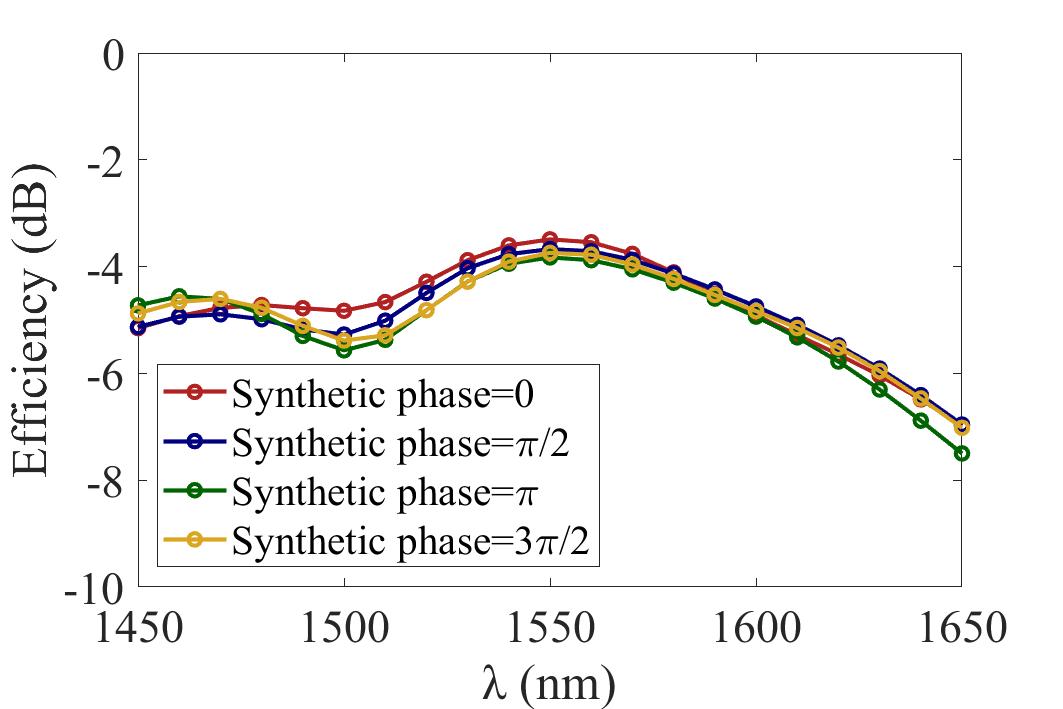


**Figure S7.** The simulated conversion efficiency of LP modes.

5. The effect of grating size on performance

Higher-order OAM can be generated by this method. In the simulation, one can see that the grating size greatly affects the mode purity and scattering efficiency. As shown in Fig. S8(a), one can see that higher-order OAM generation needs larger holographic structure size. However, when the grating size is too large, the mode purities decrease. In Fig. S8(b), the scattering efficiency becomes larger with the increase of the grating size. Therefore, there should be a trade-off between the mode purity and scattering efficiency. As shown in Fig. S8(c), OAM modes with different topological charges are generated in the simulation, which shows the intensity and phase profiles of high-order OAM modes.


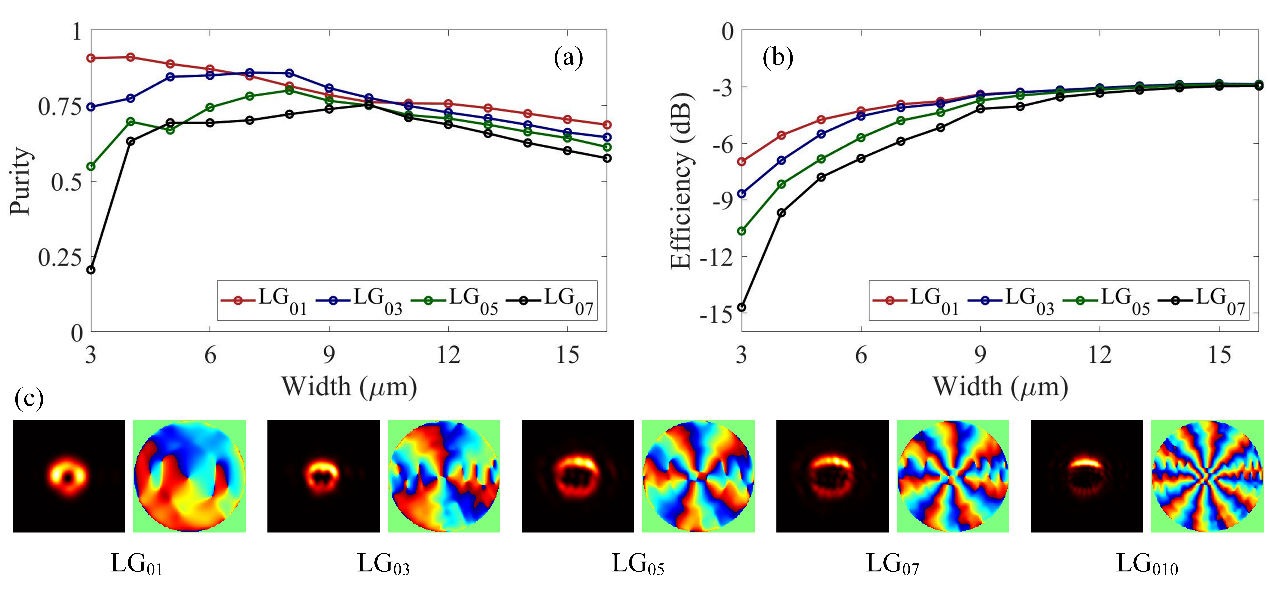


**Figure S8.** (a)(b) The calculated mode purity and scattering efficiency with the change of grating size. (c) The simulated far-field intensity and phase distributions.


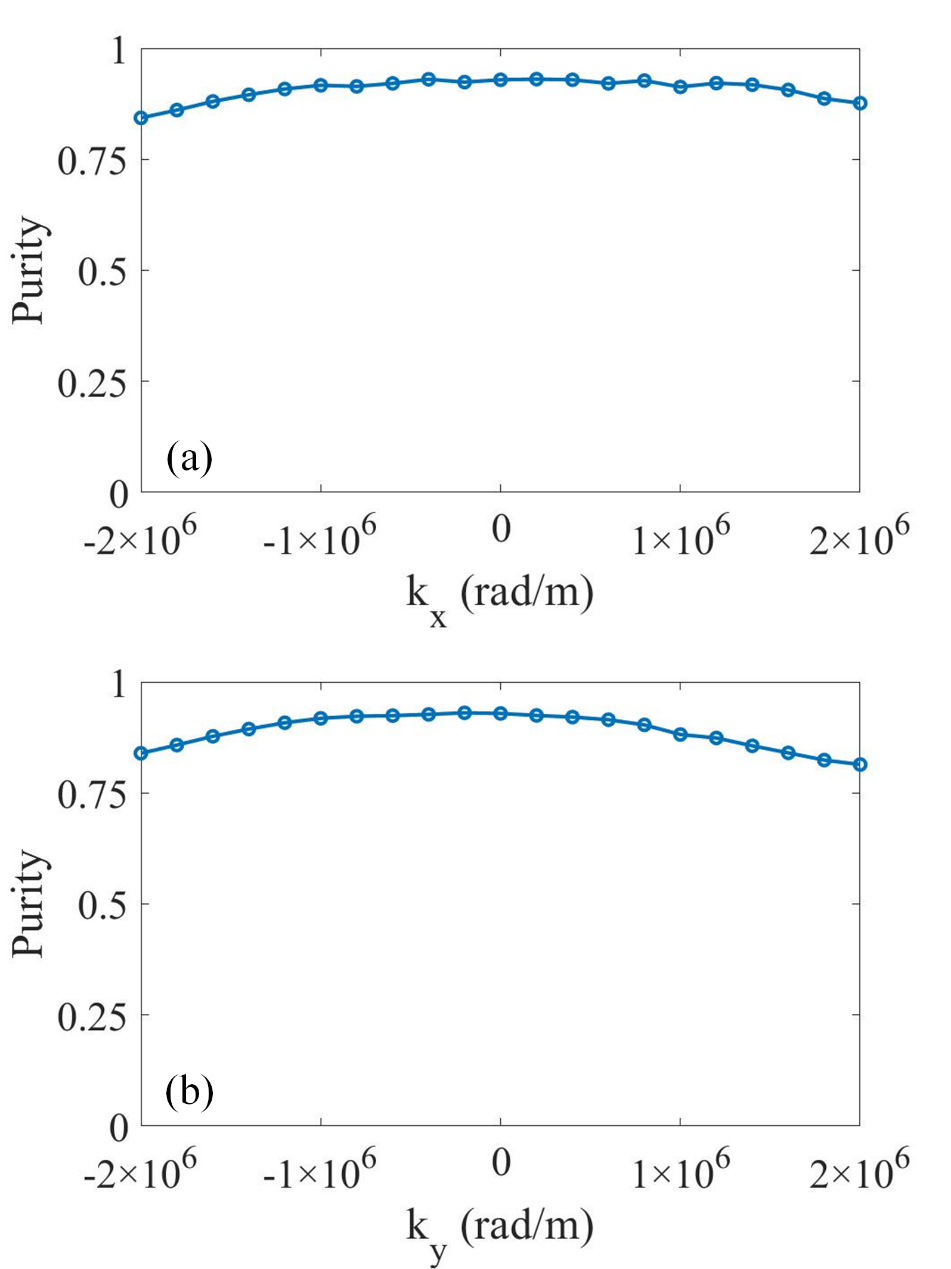


**Figure S9.** (a)(b) The calculated mode purity with the change of the phase gradients k_x_ and k_y_.

7. Experimental setup

Figure S10 shows the experimental setup for characterizing the fabricated device on a silicon platform. In the experiment, a tunable continuous-wave laser source (TSL-770) is used to measure the fabricated device over a wide wavelength range. First, the laser output is divided into two branches via a 3-dB coupler. One branch with its polarization controlled by a polarization controller (PC) is used to excite the in-plane TE_0_ mode guided in the fabricated chip-scale silicon OAM generator via an edge coupler. Then, the in-plane guided TE_0_ mode in the silicon waveguide is coupled out into free space and converted to OAM mode by the subwavelength surface structure. The output light is collimated by a ×10 objective lens, and another lens is adopted to adjust the beam size. The other branch serves as a reference Gaussian beam, which is adjusted by using a variable optical attenuator (VOA), a PC, and a collimator. The generated OAM mode and the reference Gaussian beam with the same polarization, proper relative power, and beam size are combined through a beam splitter (BS) to produce the interferograms. The wavelength tuning range of the laser is from 1480 to 1600 nm. The attenuation tuning range of the VOA is from 2 to 60 dB. An infrared camera is used to monitor the intensity profiles of generated OAM modes and their corresponding interferograms.


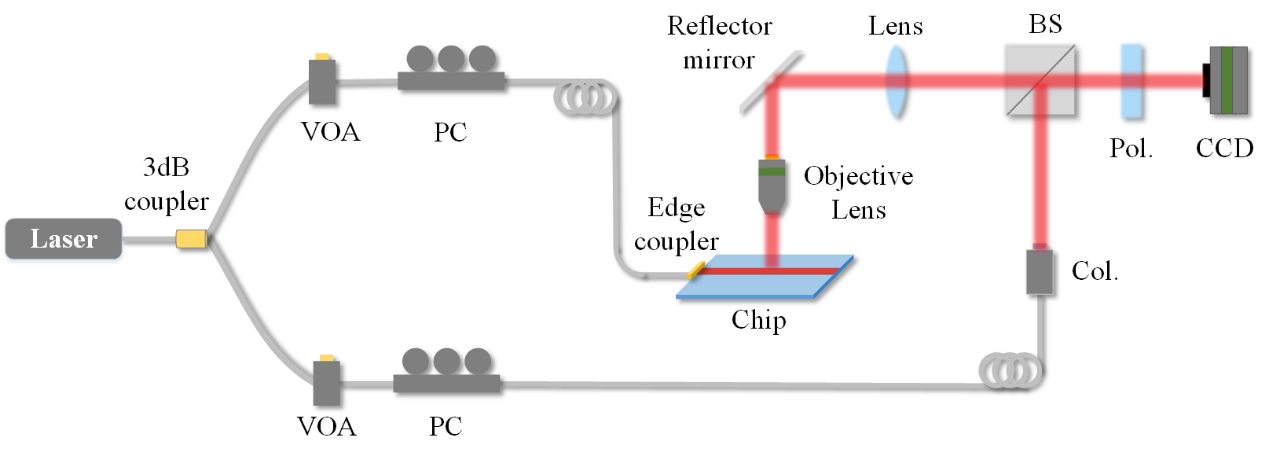


**Figure S10.** Experimental setup. PC, polarization controller; VOA, variable optical attenuator; Col., collimator; Pol., polarizer; BS, beam splitter.
